# Host genetics of HCV disease — *IL28B*

**DOI:** 10.1186/1758-2652-13-S4-O30

**Published:** 2010-11-08

**Authors:** J Fellay

**Affiliations:** 1 Institute of Microbiology and Service of Infectious Diseases, University of Lausanne, Geneva, Switzerland

## 

Co-infections with HCV and HIV are common, because both viruses share routes of transmission and establish chronic infections. The standard treatment for chronic hepatitis C, a combination of peginterferon-α and ribavirin, is poorly tolerated and only successful in about half of the treated patients. Thus, identification of accurate predictors of treatment response is highly desirable.

Several independent genome-wide association studies have recently identified human genetic variants around the *IL28B* gene (coding for IFN-λ3) that strongly associate with spontaneous clearance of HCV and with treatment success, both in HCV mono-infected and in co-infected patients. I will put these findings in perspective and discuss their practical and theoretical implications with regard to drug development, clinical trial design and clinical management of chronic HCV infection. Results of detailed genetic and functional analyses of the *IL28B* gene region will also be presented.

